# 4-(4-Ethyl­phenyl­diazen­yl)phenol

**DOI:** 10.1107/S160053680801338X

**Published:** 2008-05-10

**Authors:** Joost de Wit, Gert O. R. Alberda van Ekenstein, Gerrit ten Brinke, Auke Meetsma

**Affiliations:** a Laboratory of Polymer Chemistry, Zernike Institute for Advanced Materials, University of Groningen, NL-9747 AG Groningen, The Netherlands; b Crystal Structure Center, Chemical Physics, Zernike Institute for Advanced Materials, University of Groningen, Nijenborgh 4, NL-9747 AG Groningen, The Netherlands

## Abstract

The crystal structure of the title compound, C_14_H_14_N_2_O, determined at 100 K, shows that the mol­ecules are not planar in the solid state, in contrast to other diazene (azobenzene) derivatives. The dihedral angle between the planes of the two aromatic rings is 42.32 (7)°. The mol­ecules are linked by inter­molecular O—H⋯N hydrogen bonds, forming an infinite one-dimensional chain.

## Related literature

For related literature, see: Bowes *et al.* (2003 ▶); Brown *et al.* (1971 ▶); Burger & Ramberger (1979 ▶); Enkelmann *et al.* (1978 ▶); Kageyama *et al.* (1982 ▶, 1985 ▶, 1986 ▶); Kashino *et al.* (1979 ▶); Kocaokutgen *et al.* (2003 ▶); McWilliam *et al.* (2001 ▶); Okamoto *et al.* (1983 ▶); Okamoto & Nakano (1994 ▶); Ruokolainen *et al.* (1996 ▶, 1998 ▶, 1999 ▶); Shibaev *et al.* (2003 ▶); Soylu *et al.* (2004 ▶); Zhang *et al.* (1998 ▶).
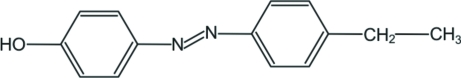


## Experimental

### 

#### Crystal data

C_14_H_14_N_2_O
*M* *_r_* = 226.28
Monoclinic, 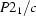

*a* = 7.5261 (9) Å
*b* = 13.4298 (15) Å
*c* = 11.6412 (13) Å
β = 97.4001 (15)°
*V* = 1166.8 (2) Å^3^
*Z* = 4
Mo *K*α radiation
μ = 0.08 mm^−1^
*T* = 100 (1) K
0.42 × 0.33 × 0.22 mm

#### Data collection

Bruker SMART APEX CCD area-detector diffractometer
Absorption correction: multi-scan (*SADABS*; Bruker, 2007 ▶) *T*_min_ = 0.956, *T*_max_ = 0.982
8700 measured reflections
2276 independent reflections
1848 reflections with *I* > 2σ(*I*)
*R*_int_ = 0.028

#### Refinement

*R*[*F* ^2^ > 2σ(*F* ^2^)] = 0.040
*wR*(*F* ^2^) = 0.107
*S* = 1.04
2276 reflections
210 parameters
All H-atom parameters refined
Δρ_max_ = 0.17 e Å^−3^
Δρ_min_ = −0.24 e Å^−3^

### 

Data collection: *SMART* (Bruker, 2007 ▶); cell refinement: *SAINT-Plus* (Bruker, 2007 ▶); data reduction: *SAINT-Plus*; program(s) used to solve structure: *SIR2004* (Burla *et al.*, 2005 ▶); program(s) used to refine structure: *SHELXL97* (Sheldrick, 2008 ▶); molecular graphics: *PLATON* (Spek, 2003 ▶) and *PLUTO* (Meetsma, 2007 ▶); software used to prepare material for publication: *PLATON*.

## Supplementary Material

Crystal structure: contains datablocks global, I. DOI: 10.1107/S160053680801338X/bg2174sup1.cif

Structure factors: contains datablocks I. DOI: 10.1107/S160053680801338X/bg2174Isup2.hkl

Additional supplementary materials:  crystallographic information; 3D view; checkCIF report

## Figures and Tables

**Table 1:** Hydrogen-bond geometry (Å, °)

*D*—H⋯*A*	*D*—H	H⋯*A*	*D*⋯*A*	*D*—H⋯*A*
O1—H1⋯N1^i^	0.91 (2)	1.98 (2)	2.8316 (15)	154.7 (17)
C3—H3⋯O1^ii^	0.987 (15)	2.585 (15)	3.3541 (17)	134.7 (11)

Symmetry codes: (i) 
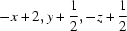
; (ii) 
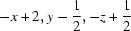
.

**Fig. 1.**
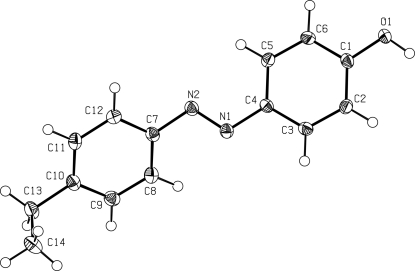
Perspective ORTEP drawing of (I) with the atom-labeling scheme of the non-hydrogen atoms. All atoms are represented by their displacement ellipsoids drawn at 50% probability level.

**Fig. 2.**
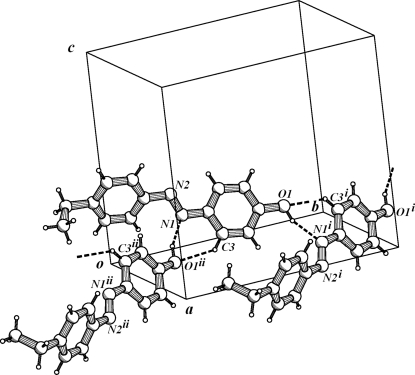
Crystal packing view of (I), showing the hydrogen bonds as dashed lines. Symmetry codes, as in Table 1.

## supplementary crystallographic information

### Comment

Azobenzenes are widely used as dyes, but also as photochemical switch using photo-isomerization by UV light to induce a conformational change from *trans* to *cis* and back. This principle has been explored extensively in order to exploit the isomerization for several applications. (Shibaev *et al.*, 2003) The presence of the phenolic moiety makes it possible to form complexes with *e.g.* poly (4-vinylpyridine) homopolymer or with poly (4-vinylpyridine) containing block copolymers. These supramolecular comb-like polymeric structures give rise to hierarchical structure-in-structure morphologies comparable with the systems with poly (4-vinylpyridine) containing block co-polymers complexed with nona- or pentadecylphenol. (Ruokolainen *et al.*, 1998, 1999) The molecular geometry of (I) and the adopted atom-numbering scheme are shown in the perspective view in Figure 1. The crystal structure is similar to several other azo compounds, (Kocaokutgen *et al.* 2003; Soylu *et al.* 2004; Zhang *et al.*, 1998) The –N1=N2- bond length is 1.2636 (16) Å, indicating a double-bond character. Regarding the azo double bond the rings are in a *trans* configuration. In contrast to many other azocompounds the benzene rings of EPAP are not coplanar.

### Experimental

To a vigorously stirred solution of 1.21 g of ethylaniline (0.01 mol) in 3 ml of water and 3 ml of concentrated hydrochloric acid, a solution of 2.8 g sodiumnitrite in 20 ml water was added drop wise while maintaining the temperature during the reaction at 0 °C. The resulting pale yellow mixture was added drop wise to a phenolate ion solution which was prepared by dissolving 0.94 g phenol (0.01 mol) and 0.84 g potassium hydroxide (0.015 mol) in 20 ml of methanol. Dichloromethane was used to extract the product from the aquatic reaction mixture, followed by 5 times washing with water. The solvent was then removed by evaporation. Subsequently the crude product was purified over a silica gel column using a dichloromethane / n-hexane mixture (3:1 *v*/*v*). The final solution was evaporated to dryness and dried further overnight in vacuum at 40°C. The yield of the bright orange crystalline solid was 61%. Single crystals of (I) suitable for the *x*-ray analysis were grown by slow evaporation from a dichloromethane solution at room temperature in air.

Analysis Differential Scanning Calorimetry (DSC, Q1000 TA instruments; 10°C/min): melting point (onset) 120°C. H-NMR (Varian VXR 300 MHz, CDCl_3_): δ p.p.m. = 7.85 (d, 2H, H-3 and H-5), 7.79 p.p.m. (d, 2H, H-3' and H-5'), 7.31 p.p.m. (d, 2*H*, H-2' and H-6'), 6.92 p.p.m. (d, 2H, H-2 and H-6), 5.09 p.p.m. (s, 1H, –OH), 2.67 p.p.m. (t, 2H, H_A_), 1.57 p.p.m. (m, 2H, –CH2-)), 1.26 p.p.m. (t, 3H, CH_3_—CH_2_). Mass (Jeol JMS 600H EI+ 70 eV): m/*z* = 226 (calculated: 226.3)

### Refinement

All hydrogen atoms were located in a difference Fourier map and refined with isotropic displacement parameters.

C—H distances spanned the range of 0.943 (16)–1.011 (18) Å, and U(H) factors, 0.054 (6) to 0.019 (4) Å^-2^.

### Crystal data

**Table:**

C_14_H_14_N_2_O	*F*_000_ = 480
*M**_r_* = 226.28	*D*_x_ = 1.288 Mg m^−^^3^
Monoclinic, *P*2_1_/*c*	Mo *K*α radiation λ = 0.71073 Å
Hall symbol: -P 2ybc	Cell parameters from 3059 reflections
*a* = 7.5261 (9) Å	θ = 3.0–27.5º
*b* = 13.4298 (15) Å	µ = 0.08 mm^−^^1^
*c* = 11.6412 (13) Å	*T* = 100 (1) K
β = 97.4001 (15)º	Block, orange
*V* = 1166.8 (2) Å^3^	0.42 × 0.33 × 0.22 mm
*Z* = 4	

### Data collection

**Table:**

Bruker SMART APEX CCD area-detector diffractometer	2276 independent reflections
Monochromator: parallel mounted graphite	1848 reflections with *I* > 2σ(*I*)
Detector resolution: 66.06 pixels mm^-1^	*R*_int_ = 0.028
*T* = 100(1) K	θ_max_ = 26.0º
φ and ω scans	θ_min_ = 3.0º
Absorption correction: multi-scan(SADABS; Bruker, 2007)	*h* = −9→9
*T*_min_ = 0.956, *T*_max_ = 0.982	*k* = −16→15
8700 measured reflections	*l* = −14→14

### Refinement

**Table:**

Refinement on *F*^2^	Secondary atom site location: structure-invariant direct methods
Least-squares matrix: full	Hydrogen site location: difference Fourier map
*R*[*F*^2^ > 2σ(*F*^2^)] = 0.040	All H-atom parameters refined
*wR*(*F*^2^) = 0.107	*w* = 1/[σ^2^(*F*_o_^2^) + (0.0608*P*)^2^ + 0.2293*P*] where *P* = (*F*_o_^2^ + 2*F*_c_^2^)/3
*S* = 1.04	(Δ/σ)_max_ < 0.001
2276 reflections	Δρ_max_ = 0.17 e Å^−^^3^
210 parameters	Δρ_min_ = −0.24 e Å^−^^3^
Primary atom site location: heavy-atom method	Extinction correction: none

### Special details

**Table:**

Experimental. The final unit cell was obtained from the xyz centroids of 3059 reflections after integration using the SAINTPLUS software package (Bruker, 2007).Reduced cell calculations did not indicate any higher metric lattice symmetry and examination of the final atomic coordinates of the structure did not yield extra symmetry elements (Spek, 1988; Le Page 1987, 1988)
Geometry. Bond distances, angles *etc*. have been calculated using the rounded fractional coordinates. All su's are estimated from the variances of the (full) variance-covariance matrix. The cell e.s.d.'s are taken into account in the estimation of distances, angles and torsion angles
Refinement. Refinement of *F*^2^ against ALL reflections. The weighted *R*-factor *wR* and goodness of fit *S* are based on *F*^2^, conventional *R*-factors *R* are based on *F*, with *F* set to zero for negative *F*^2^. The threshold expression of *F*^2^ > σ(*F*^2^) is used only for calculating *R*-factors(gt) *etc*. and is not relevant to the choice of reflections for refinement. *R*-factors based on *F*^2^ are statistically about twice as large as those based on *F*, and *R*- factors based on ALL data will be even larger.

### Fractional atomic coordinates and isotropic or equivalent isotropic displacement parameters (Å^2^)

**Table:**

	*x*	*y*	*z*	*U*_iso_*/*U*_eq_	
O1	1.04406 (14)	0.48064 (7)	0.32923 (9)	0.0263 (3)	
N1	0.80788 (15)	0.08833 (8)	0.34477 (9)	0.0188 (3)	
N2	0.75956 (15)	0.06025 (8)	0.43960 (10)	0.0199 (3)	
C1	0.98453 (18)	0.38503 (10)	0.32795 (11)	0.0193 (4)	
C2	0.99777 (18)	0.31861 (10)	0.23708 (12)	0.0201 (4)	
C3	0.93839 (18)	0.22132 (10)	0.24543 (12)	0.0190 (4)	
C4	0.86411 (17)	0.18959 (10)	0.34290 (11)	0.0175 (4)	
C5	0.84621 (18)	0.25739 (10)	0.43237 (12)	0.0190 (4)	
C6	0.90547 (18)	0.35397 (10)	0.42467 (12)	0.0204 (4)	
C7	0.69280 (17)	−0.03918 (10)	0.43646 (12)	0.0190 (4)	
C8	0.59745 (19)	−0.08196 (11)	0.33765 (12)	0.0223 (4)	
C9	0.53750 (19)	−0.17915 (11)	0.34156 (13)	0.0232 (4)	
C10	0.57357 (18)	−0.23637 (10)	0.44256 (12)	0.0214 (4)	
C11	0.66447 (19)	−0.19152 (11)	0.54092 (13)	0.0228 (4)	
C12	0.72082 (19)	−0.09323 (11)	0.53944 (12)	0.0216 (4)	
C13	0.5212 (2)	−0.34494 (11)	0.44295 (14)	0.0260 (5)	
C14	0.6554 (2)	−0.41051 (12)	0.39094 (16)	0.0298 (5)	
H1	1.096 (3)	0.4963 (14)	0.2651 (19)	0.054 (6)*	
H2	1.0497 (19)	0.3401 (11)	0.1707 (13)	0.025 (4)*	
H3	0.9523 (19)	0.1728 (11)	0.1835 (13)	0.022 (4)*	
H5	0.7933 (19)	0.2348 (10)	0.4993 (13)	0.019 (4)*	
H6	0.894 (2)	0.4036 (11)	0.4876 (13)	0.023 (4)*	
H8	0.569 (2)	−0.0420 (12)	0.2682 (14)	0.026 (4)*	
H9	0.472 (2)	−0.2076 (11)	0.2751 (14)	0.025 (4)*	
H11	0.690 (2)	−0.2308 (11)	0.6131 (13)	0.023 (4)*	
H12	0.781 (2)	−0.0609 (11)	0.6080 (14)	0.027 (4)*	
H13	0.515 (2)	−0.3675 (13)	0.5252 (16)	0.043 (5)*	
H13'	0.401 (2)	−0.3550 (12)	0.3973 (15)	0.038 (5)*	
H14	0.779 (3)	−0.4016 (13)	0.4330 (16)	0.047 (5)*	
H14'	0.662 (2)	−0.3941 (13)	0.3112 (17)	0.041 (5)*	
H14"	0.624 (2)	−0.4825 (13)	0.3936 (15)	0.038 (5)*	

### Atomic displacement parameters (Å^2^)

**Table:**

	*U*^11^	*U*^22^	*U*^33^	*U*^12^	*U*^13^	*U*^23^
O1	0.0395 (6)	0.0160 (5)	0.0260 (6)	−0.0050 (4)	0.0138 (5)	−0.0013 (4)
N1	0.0182 (6)	0.0180 (6)	0.0204 (6)	0.0009 (4)	0.0032 (5)	0.0016 (5)
N2	0.0207 (6)	0.0169 (6)	0.0224 (6)	0.0011 (4)	0.0042 (5)	0.0009 (5)
C1	0.0215 (7)	0.0149 (7)	0.0211 (7)	0.0007 (5)	0.0017 (5)	0.0019 (5)
C2	0.0220 (7)	0.0201 (7)	0.0186 (7)	0.0004 (5)	0.0046 (5)	0.0025 (6)
C3	0.0197 (7)	0.0176 (7)	0.0195 (7)	0.0015 (5)	0.0020 (5)	−0.0015 (6)
C4	0.0173 (7)	0.0148 (7)	0.0202 (7)	0.0009 (5)	0.0017 (5)	0.0000 (5)
C5	0.0196 (7)	0.0195 (7)	0.0184 (7)	0.0020 (5)	0.0042 (5)	0.0022 (5)
C6	0.0241 (7)	0.0175 (7)	0.0201 (7)	0.0018 (5)	0.0044 (6)	−0.0026 (5)
C7	0.0180 (7)	0.0155 (7)	0.0244 (7)	0.0017 (5)	0.0067 (5)	−0.0003 (5)
C8	0.0214 (7)	0.0216 (8)	0.0240 (7)	0.0006 (6)	0.0037 (6)	0.0038 (6)
C9	0.0212 (7)	0.0235 (8)	0.0248 (8)	−0.0027 (6)	0.0029 (6)	−0.0026 (6)
C10	0.0173 (7)	0.0185 (7)	0.0298 (8)	−0.0005 (5)	0.0082 (6)	−0.0005 (6)
C11	0.0231 (7)	0.0205 (8)	0.0253 (8)	0.0012 (6)	0.0048 (6)	0.0059 (6)
C12	0.0220 (8)	0.0219 (8)	0.0210 (7)	−0.0004 (6)	0.0034 (6)	−0.0001 (6)
C13	0.0258 (8)	0.0192 (8)	0.0341 (9)	−0.0036 (6)	0.0078 (7)	0.0008 (6)
C14	0.0288 (9)	0.0209 (8)	0.0398 (10)	−0.0010 (6)	0.0044 (7)	−0.0051 (7)

### Geometric parameters (Å, °)

**Table:**

O1—C1	1.3594 (17)	C10—C13	1.511 (2)
O1—H1	0.91 (2)	C11—C12	1.387 (2)
N1—N2	1.2636 (16)	C13—C14	1.523 (2)
N1—C4	1.4252 (17)	C2—H2	0.954 (15)
N2—C7	1.4255 (17)	C3—H3	0.987 (15)
C1—C6	1.4030 (19)	C5—H5	0.968 (15)
C1—C2	1.3971 (19)	C6—H6	1.002 (15)
C2—C3	1.3884 (19)	C8—H8	0.971 (16)
C3—C4	1.3946 (19)	C9—H9	0.943 (16)
C4—C5	1.4029 (19)	C11—H11	0.990 (15)
C5—C6	1.3783 (19)	C12—H12	0.968 (16)
C7—C8	1.398 (2)	C13—H13	1.011 (18)
C7—C12	1.394 (2)	C13—H13'	0.998 (16)
C8—C9	1.384 (2)	C14—H14	1.00 (2)
C9—C10	1.402 (2)	C14—H14'	0.96 (2)
C10—C11	1.393 (2)	C14—H14"	0.997 (17)
			
O1···N1^i^	2.8316 (15)	C8···H2^iii^	2.868 (15)
O1···C3^i^	3.3541 (17)	C9···H14'	3.069 (17)
O1···H3^i^	2.585 (15)	C10···H5^v^	2.926 (15)
O1···H6^ii^	2.632 (15)	C13···H5^v^	2.942 (14)
N1···O1^iii^	2.8316 (15)	C14···H8^x^	2.932 (16)
N1···H8	2.584 (16)	H1···N1^i^	1.98 (2)
N1···H1^iii^	1.98 (2)	H1···N2^i^	2.88 (2)
N2···H5	2.449 (14)	H1···C3^i^	3.034 (19)
N2···H1^iii^	2.88 (2)	H1···C4^i^	2.92 (2)
C2···C8^i^	3.536 (2)	H1···C7^i^	3.04 (2)
C3···O1^iii^	3.3541 (17)	H1···C8^i^	2.93 (2)
C4···C12^iv^	3.494 (2)	H2···C7^i^	2.926 (15)
C5···C13^v^	3.488 (2)	H2···C8^i^	2.868 (15)
C7···C7^v^	3.5810 (19)	H3···O1^iii^	2.585 (15)
C8···C2^iii^	3.536 (2)	H3···C5^vi^	3.076 (15)
C12···C4^iv^	3.494 (2)	H3···C6^vi^	3.010 (15)
C13···C5^v^	3.488 (2)	H5···N2	2.449 (14)
C2···H5^vi^	3.074 (15)	H5···C10^v^	2.926 (15)
C2···H11^iv^	2.983 (15)	H5···C13^v^	2.942 (14)
C3···H13'^vii^	3.040 (16)	H5···C2^viii^	3.074 (15)
C3···H11^iv^	3.060 (15)	H5···C3^viii^	2.991 (15)
C3···H5^vi^	2.991 (15)	H6···O1^ii^	2.632 (15)
C3···H1^iii^	3.034 (19)	H8···N1	2.584 (16)
C4···H1^iii^	2.92 (2)	H8···C14^vii^	2.932 (16)
C4···H9^vii^	3.049 (15)	H9···C4^x^	3.049 (15)
C5···H3^viii^	3.076 (15)	H11···C2^iv^	2.983 (15)
C6···H14^iv^	2.79 (2)	H11···C3^iv^	3.060 (15)
C6···H3^viii^	3.010 (15)	H13'···C3^x^	3.040 (16)
C6···H14"^ix^	3.040 (17)	H14···C6^iv^	2.79 (2)
C7···H2^iii^	2.926 (15)	H14'···C9	3.069 (17)
C7···H1^iii^	3.04 (2)	H14"···C6^xi^	3.040 (17)
C8···H1^iii^	2.93 (2)		
			
C1—O1—H1	112.6 (12)	C2—C3—H3	120.3 (9)
N2—N1—C4	114.68 (10)	C4—C3—H3	118.9 (9)
N1—N2—C7	113.41 (11)	C4—C5—H5	118.9 (8)
O1—C1—C6	116.44 (12)	C6—C5—H5	121.2 (8)
C2—C1—C6	119.79 (12)	C1—C6—H6	118.3 (9)
O1—C1—C2	123.77 (12)	C5—C6—H6	121.3 (9)
C1—C2—C3	119.53 (13)	C7—C8—H8	119.5 (10)
C2—C3—C4	120.72 (13)	C9—C8—H8	120.7 (9)
N1—C4—C3	117.06 (11)	C8—C9—H9	119.6 (9)
C3—C4—C5	119.51 (12)	C10—C9—H9	119.4 (9)
N1—C4—C5	123.43 (12)	C10—C11—H11	119.2 (9)
C4—C5—C6	119.98 (13)	C12—C11—H11	119.4 (9)
C1—C6—C5	120.41 (13)	C7—C12—H12	118.6 (9)
N2—C7—C8	123.21 (12)	C11—C12—H12	121.9 (9)
C8—C7—C12	119.88 (13)	C10—C13—H13	109.6 (10)
N2—C7—C12	116.87 (12)	C10—C13—H13'	110.4 (9)
C7—C8—C9	119.70 (13)	C14—C13—H13	108.5 (9)
C8—C9—C10	121.07 (13)	C14—C13—H13'	108.2 (10)
C9—C10—C13	120.62 (13)	H13—C13—H13'	108.3 (13)
C11—C10—C13	121.04 (13)	C13—C14—H14	111.0 (11)
C9—C10—C11	118.30 (13)	C13—C14—H14'	111.8 (10)
C10—C11—C12	121.32 (13)	C13—C14—H14"	112.1 (9)
C7—C12—C11	119.57 (13)	H14—C14—H14'	106.5 (14)
C10—C13—C14	111.73 (12)	H14—C14—H14"	108.1 (14)
C1—C2—H2	119.8 (9)	H14'—C14—H14"	107.1 (14)
C3—C2—H2	120.7 (9)		
			
C4—N1—N2—C7	176.04 (11)	C4—C5—C6—C1	−0.3 (2)
N2—N1—C4—C3	172.74 (12)	N2—C7—C8—C9	179.83 (13)
N2—N1—C4—C5	−8.05 (18)	C12—C7—C8—C9	−2.5 (2)
N1—N2—C7—C8	−33.70 (18)	N2—C7—C12—C11	−177.78 (13)
N1—N2—C7—C12	148.57 (12)	C8—C7—C12—C11	4.4 (2)
O1—C1—C2—C3	177.87 (13)	C7—C8—C9—C10	−1.2 (2)
C6—C1—C2—C3	−2.4 (2)	C8—C9—C10—C11	2.9 (2)
O1—C1—C6—C5	−178.04 (12)	C8—C9—C10—C13	−174.80 (13)
C2—C1—C6—C5	2.2 (2)	C9—C10—C11—C12	−1.0 (2)
C1—C2—C3—C4	0.7 (2)	C13—C10—C11—C12	176.74 (13)
C2—C3—C4—N1	−179.53 (12)	C9—C10—C13—C14	80.03 (17)
C2—C3—C4—C5	1.2 (2)	C11—C10—C13—C14	−97.60 (17)
N1—C4—C5—C6	179.38 (12)	C10—C11—C12—C7	−2.7 (2)
C3—C4—C5—C6	−1.4 (2)		

Symmetry codes: (i) −*x*+2, *y*+1/2, −*z*+1/2; (ii) −*x*+2, −*y*+1, −*z*+1; (iii) −*x*+2, *y*−1/2, −*z*+1/2; (iv) −*x*+2, −*y*, −*z*+1; (v) −*x*+1, −*y*, −*z*+1; (vi) *x*, −*y*+1/2, *z*−1/2; (vii) −*x*+1, *y*+1/2, −*z*+1/2; (viii) *x*, −*y*+1/2, *z*+1/2; (ix) *x*, *y*+1, *z*; (x) −*x*+1, *y*−1/2, −*z*+1/2; (xi) *x*, *y*−1, *z*.

### Hydrogen-bond geometry (Å, °)

**Table:**

*D*—H···*A*	*D*—H	H···*A*	*D*···*A*	*D*—H···*A*
O1—H1···N1^i^	0.91 (2)	1.98 (2)	2.8316 (15)	154.7 (17)
C3—H3···O1^iii^	0.987 (15)	2.585 (15)	3.3541 (17)	134.7 (11)

Symmetry codes: (i) −*x*+2, *y*+1/2, −*z*+1/2; (iii) −*x*+2, *y*−1/2, −*z*+1/2.
